# Putting Brain Training to the Test in the Workplace: A Randomized, Blinded, Multisite, Active-Controlled Trial

**DOI:** 10.1371/journal.pone.0059982

**Published:** 2013-03-28

**Authors:** Catherine Borness, Judith Proudfoot, John Crawford, Michael Valenzuela

**Affiliations:** 1 School of Psychiatry, Faculty of Medicine, University of New South Wales, Sydney, New South Wales, Australia; 2 Black Dog Institute, University of New South Wales, Sydney, New South Wales, Australia; 3 Centre for Healthy Brain Ageing, Faculty of Medicine, University of New South Wales, Sydney, New South Wales, Australia; 4 Regenerative Neuroscience Group, Brain & Mind Research Institute, University of Sydney, Sydney, New South Wales, Australia; University Of São Paulo, Brazil

## Abstract

**Background:**

Cognitive training (CT) is effective at improving cognitive outcomes in children with and without clinical impairment as well as older individuals. Yet whether CT is of any preventative health benefit to working age adults is controversial. Our objective was therefore to investigate the real-world efficacy of CT in the workplace, involving employees from across the working-age spectrum and addressing many of the design issues that have limited trials to date.

**Methods and Findings:**

135 white collar employees of a large Australian public sector organization were randomised to either 16 weeks (20 minutes three times per week) of online CT or an active control (AC) program of equal length and structure. Cognitive, wellbeing and productivity outcome measures were analysed across three timepoints: baseline, immediately after training and 6 months post-training. CT effects on cognitive outcomes were limited, even after planned subgroup analyses of cognitive capacity and age. Unexpectedly, we found that our AC condition, which comprised viewing short documentaries about the natural world, had more impact. Compared to the CT group, 6 months after the end of training, those in the AC group experienced a significant increase in their self-reported Quality of Life (Effect Size *g* = .34 vs −.15; TIME×GROUP *p* = .003), decrease in stress levels (*g* = .22 vs −.19; TIME x GROUP *p* = .03), and overall improvement in Psychological Wellbeing (*g* = .32 vs −.06; TIME×GROUP *p* = .02).

**Conclusions:**

CT does not appear to positively impact cognition or wellbeing amongst white collar office workers; however, short time-out respite activities may have value in the promotion of psychological wellbeing. Given looming challenges to workplace productivity, further work-based interventional research targeting employee mental health is recommended.

**Trial Registration:**

This trial was registered with the Australian New Zealand Clinical Trials Registry: ACTRN12610000604000 (http://www.anzctr.org.au/TrialSearch.aspx).

## Introduction

Demographic ageing of modern and developing nations as well as the rising incidence of mental health disorders represent major threats to workforce productivity in the coming decades [1]. Advanced age is the single greatest risk factor for cognitive decline [2], with each decade after 20 years of age associated with an 8% reduction in memory function [3], 7% reduction in frontal-executive function and 8% reduction in attentional capacity [4]. In addition, the Australian Productivity Commission has found that of the six major health conditions, mental illness predicts the lowest likelihood of workforce participation [5]. Depression in particular is detrimental to job performance [6] and has further negative effects on cognitive ability [7]. Moreover, the interaction of advanced age and depression is particularly potent, increasing the risk of mild cognitive impairment (MCI) and rapid age-related cognitive decline [8]. Taken together, these population-level changes are already placing pressure on workforce productivity.

At the same time, neuroscientific studies of cognition and mental health are having an influence on organizational behaviour and human resource management [9], [10]. Computerized cognitive training (CT), or ‘brain training’, has received much attention in the clinical environment, with evidence of improved symptoms in depression [11], [12], schizophrenia [13], [14], Attention Deficit Hyperactivity Disorder [15], MCI and dementia [16], [17]. Furthermore, long term benefits after the cessation of training have also been reported [14], [18]. However, efficacy of CT in healthy working age individuals is contested. Owen et al (2010) questioned the impact of CT on cognitive ability in healthy adults and the transfer of training to non-trained tasks [19], but this study has been criticised on a number of methodological grounds [20], [21]. While CT has been reported to be vocationally effective, for example, targeted single domain CT improves motor control in surgeons [22] as well as pilots’ flight performance through enhanced attentional control [23], it remains untested in the workplace as a human resource intervention for the prevention or maintenance of cognitive capacity or mental health outcomes.

Our objective was therefore to investigate the real-world efficacy of CT in the workplace, involving employees from across the full working age spectrum and addressing many of the design issues that have limited CT studies to date [24]. We randomized participants to either 16 weeks of online CT or an online AC program of equal length and structure. Outcomes included various cognitive, wellbeing and productivity measures which were collected pre-training, immediately post-training and 6 months post-training. We also planned an *a priori* subgroup analysis based on a split of baseline subjects into low- and high- cognitive capacity. Our specific aims were to test whether computerized cognitive brain training would, i) increase cognitive abilities vital to effective and efficient workplace performance, ii) augment positive psychological measures of wellbeing and quality of life, and iii) improve objective measures of workplace productivity.

## Methods

The protocol for this trial and supporting CONSORT checklist are available as supporting information (see **Checklist S1** and **Protocol S1**).

### Ethics Statement

This research was approved by the University of New South Wales’ Human Research Ethics Committee. Written informed consent was obtained from each volunteer. By giving Informed Consent, participants were also agreeing that they did not meet any of the exclusion criteria.

### Research Design

This study was a 1∶1 randomized, active controlled, single-blind, multi-centre intervention trial with longitudinal follow-up 6 months post intervention, approximately 10 months since the start of the trial.

### Participants

**Figure 1:**
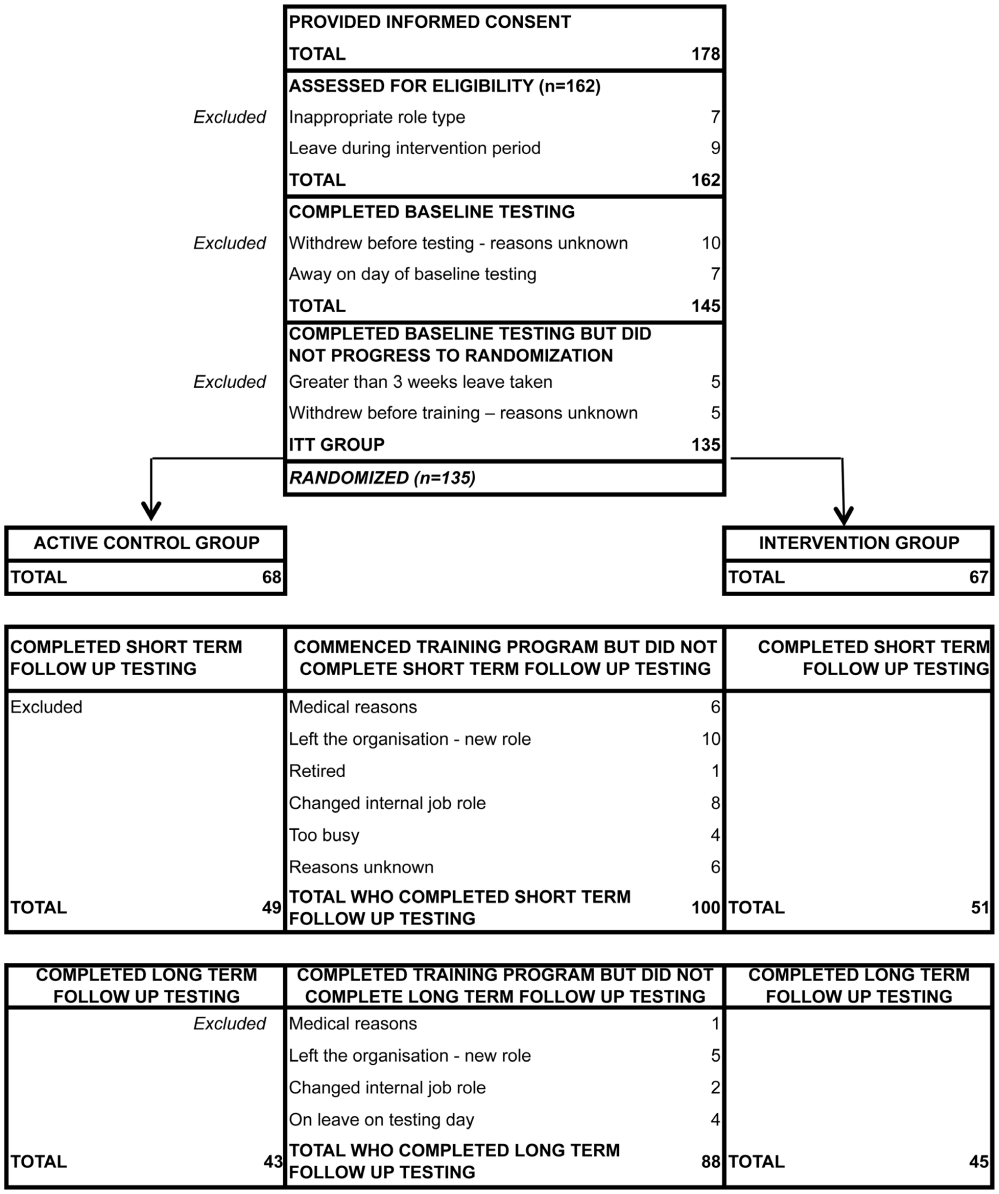
Consort Flow Diagram.

Our sample consisted of full time and part time (working a minimum of 3 days per week) staff from an Australian national public service organization aged between 18 and 65 and employed for a minimum of 6 months by that organization at one of six different office locations around Australia. Any volunteers who were currently using any other form of computer based brain training, were planning to take 3 or more weeks’ leave during the intervention period, or had any clinical diagnoses or treatment for mood disorders or drug dependency, were excluded from the study. While 178 employees initially volunteered for the study, the final intention-to-treat (ITT) sample comprised 135 participants. See **Figure 1** **: Consort Flow Diagram.**

Based on power analyses conducted in our pilot study, a sample size of 220 (110 for each condition) was ideal however the final sample did not reach this number. (See **Protocol S1** for more information on power analysis). recruited sample (N = 178) did not differ significantly from the ITT (N = 135) group on any demographic variables. In addition, the ITT group (N = 135) and the per protocol completers (PPC) group (N = 88), that is, those participants who completed the full assessment battery on the 3 occasions (baseline, short term and long term follow up) did not differ significantly on any demographic variables, nor on baseline performance outcome measures. See **Table 1**: **Demographic and Baseline Data**.

**Table 1:** Demographic and Baseline Data.

	ITT (N = 135)		PPC (N = 88)	
DEMOGRAPHIC DATA	Mean (SD)		Mean (SD)	
Age at baseline testing (years)	41.3(13.1)		41.8 (13.0)	
	Range: 19.7–63.6		Range: 19.7–63.3	
Years of Education	13.7 (2.4)		13.6 (2.4)	
Gender	63.7% female		63.6% female	
**COGNITIVE MEASURES**	**Mean (SD)**		**Mean (SD)**	
Matrix Reasoning (/26)	14.7 (4.5)		14.9 (4.4)	
COWAT (unlimited maximum score)	34.4 (8.6)		34.6 (8.5)	
Stroop Level 1^#^	20.6 (5.2)		20.5 (4.5)	
Stroop Level 2^#^	22.7 (5.2)		22.7 (5.2)	
Stroop Level 3^#^	20.7 (7.5)		20.7 (7.9)	
SIPS Level 1^#^	18.8 (3.2)		18.8 (3.2)	
SIPS Level 2^#^	11.3 (3.0)		11.0 (3.1)	
SIPS Level 3^#^	7.7(3.0)		7.7 (2.8)	
Visual Spatial Orientation (%)	74.2 (20.1)		76.3 (18.8)	
Verbal Memory – Total Accuracy (%)	92.2 (12.8)		93.6 (9.4)	
Delayed Verbal Memory–Total Accuracy (%)	91.1 (15.0)		93.4 (8.9)	
Non Verbal Memory – Total Accuracy (%)	78.6 (20.8)		79.4 (20.3)	
Delayed Non Verbal Memory–Total Accuracy (%)	84.4 (21.1)		85.5 (20.1)	
VSCPUT (correct responses per minute)	30.8 (8.1)		30.7 (7.6)	
VSCRTC (correct responses in seconds)	2.1 (0.5)		2.0 (0.5)	
DATIRTC (correct responses in seconds)	0.3 (0.2)		0.3 (0.1)	
DIFSCPUT (correct responses per minute)	−1.0 (7.8)		−0.6 (8.3)	
DIFSCRTC (correct response in seconds)	−0.2 (0.6)		−0.2 (0.6)	
DIFINDRTC (correct responses in seconds)	0.3 (0.5)		0.3 (0.5)	
**PSYCHOLOGICAL WELLBEING MEASURES**	**Mean (SD)**		**Mean (SD)**	
Quality Of Life Scale (/105)	76.2 (8.8)		76.4 (8.9)	
Job Satisfaction Scale (/105)	69.4 (13.9)		71.2 (12.3)	
Intention To Quit (/21)	11.5 (4.5)		11.1 (4.5)	
Professional Self Esteem Scale (/7)	5.4 (0.8)		5.4 (0.8)	
SPWB: Autonomy (/54)	38.9 (6.6)		38.9 (6.4)	
SPWB: Environmental Mastery (/54)	39.6 (6.5)		39.8 (6.6)	
SPWB: Personal Growth (/54)	42.8 (6.1)		42.9 (6.0)	
SPWB: Positive Personal Relations (/54)	41.1 (7.0)		41.3 (6.7)	
SPWB: Purpose in Life (/54)	39.4 (5.5)		39.4 (5.5)	
SPWB: Self Acceptance (/54)	37.7 (7.3)		38.1 (7.3)	
DASS42: Depression (/42)	7.0 (6.6)		6.2 (6.1)	
DASS42: Anxiety (/42)	5.5 (6.1)		5.2 (5.5)	
DASS42: Stress (/42)	10.9 (7.9)		10.4 (7.1)	
**PRODUCTIVITY MEASURES^1^**	**N**	**Mean (SD)**	**N**	**Mean (SD)**
Average Handling Time Outbound	52	31.1 (36.7)	36	28.9 (22.6)
Average Handling Time Outbound – Level of Contribution (/5)	51	3.2 (1.3)	35	3.3 (1.4)
Conversion Rate Outbound (%)	50	51.3 (27.6)	36	51.5 (29.8)
Conversion Rate Outbound - Level of Contribution (/5)	42	4.5 (0.9)	29	4.5 (0.9)
Kept Rate Outbound (%)	26	19.3 (14.9)	18	18.6 (12.3)
Kept Rate Outbound - Level of Contribution (/5)	26	3.4 (0.9)	18	3.2 (1.0)
Kept Rate Inbound (%)	23	42.3 (19.5)	18	42.7 (11.7)
Kept Rate Inbound - Level of Contribution (/5)	23	2.9 (0.9)	18	2.9 (0.7)
Quality (/5)	49	2.5 (0.6)	36	2.8 (0.5)

LEGEND.

#Composite Score = (Accuracy/RT)*100, Level of difficulty increases from 1 to 3.

SIPS: Staged Information Processing Speed.

VSCPUT: Visual Sequence Comparison Thruput.

VSCRTC: Visual Sequence Comparison Median Response Time DATIRTC – Divided Attention Indicator Alone Median Response Time.

DIFSCPUT: Difference in Sequence Comparison Alone and Dual Thruput, i.e. DATSCPUT - VSCPUT.

DIFSCRTC: Difference in Sequence Comparison Alone and Dual Median Response Times.

DIFINDRTC: Difference Between Divided Attention Indicator Alone and combined with Visual Sequence Comparison, i.e. DATDRTC – DATIRTC.

SPWB: Scales of Psychological Well Being.

DASS42: Depression Anxiety & Stress Scales (42 items).

Average Handling Time: the average time taken to complete an activity, including documentation and review work.

Conversion Rate: conversion of actions to effective outcomes, a measure of how quickly the collections officer is turning over their cases.

Kept Rate: a measure of the % of payment arrangements that are adhered to in a defined period of time.

Quality: the overall grading of an IQF (internal quality framework) assessment.

Outbound: making telephone calls to clients.

Inbound: attending to written correspondence.

Level of Contribution (LOC):rating between 1 and 5 where 1 is Unsatisfactory and 5 is Exceptional.

Note 1. Productivity measures were provided by the work organisation, as opposed to other measures collected by the research team. There was hence more missing data across this set of outcome measures than others.

### Cognitive Training & Active Control

CT comprised 36 HappyNeuron (Scientific Brain Training, Lyon, France) [25] exercises across the domains of memory, attention, language, executive function and visuospatial abilities delivered online to each worksite using the *Spark!*™ software system (The Brain Department Pty Ltd, Sydney Australia). During each 20 minute training session, subjects completed a number of exercises from across a range of these domains, and were gradually challenged by exercises of greater cognitive demand tailored to their abilities, facilitated by the program’s in-built algorithms. The AC condition consisted of viewing a series of general interest videos about the natural environment (National Geographic) and answering related multiple choice questions delivered via an online survey. Both interventions ran for 16 weeks with 3 sessions per week (20 minutes per session) and were matched for duration, level of audio and visual stimulus and mode of delivery (online and directly to the participant’s regular work computer).

### Outcome Measures

Outcome measures were collected and analysed by an organizational psychologist who remained blind to the training status of participants. All outcomes were measured at Baseline, immediately after the initial 16 week period of training (Short Term Follow Up), and then 6 months after the end of training (Long Term Follow Up). They covered cognitive, psychological wellbeing and productivity outcomes. Cognitive measures were independent of the CT intervention to reduce possible practice effects. Primary and secondary outcome measures are described in **Supporting Information S1.**

### Statistical Methods

Initial analyses were run on PPC followed by ITT using a repeated measures approach. Primary outcomes were considered separately within each group (i.e. cognitive, wellbeing and productivity). Both univariate and multivariate analyses accounting for multiple comparisons were conducted. Further statistical details are provided in **Supporting Information S1.**

## Results

All reported outcomes are based on PPC and replicated through imputation techniques based on ITT analyses. Baseline analysis of our ITT population highlighted that in comparison to the general population, this sample was equally competent across all cognitive measures except the COWAT, where they performed less effectively than their age-matched comparison group (z = −1.23) [26]. This population also had similar levels of subjective psychological wellbeing to the general population, with the exception of a higher level of self-reported Professional Self Esteem (z = +1.65) and a lower level of self-reported Personal Growth (z = −1.14), a subscale of the Scales of Psychological Wellbeing [27].

### Compliance and Subjective Feedback

Training compliance rates between the CT and AC groups were equivalent at 81.6% and 82.8% respectively. Survey data post training indicated that participants found both conditions highly engaging. While this study achieved a compliance rate above 80% for both conditions, it also identified two key user issues that impacted upon full compliance: lack of time and workplace distractions.

### Effect of Cognitive Training on Cognition

#### On completion of training

**Figure 2:**
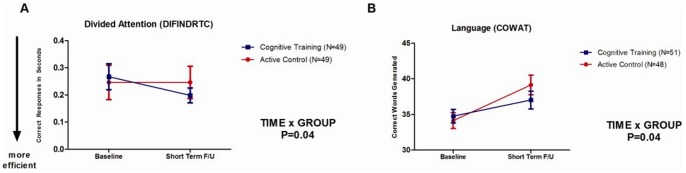
Short term effects of CT on Divided Attention and Language. Error bars represent SEMs. P-value is for TIME x TRAINING GROUP interaction.

There were 2 significant post-training TIME x TRAINING GROUP interactions for cognitive measures of divided attention and language. A Hedge’s effect size [28] of *g* = .29 was found for the CT group on one of the divided attention response time tasks (DIFINDRTC) compared to an effect size of.01 for the AC group (TIME x GROUP: F(1,90) = 4.40; *p* = .04) immediately post intervention. Those who completed CT became faster at the task, while little or no improvement was observed in the AC group on this measure. However, this was no longer significant after correction for multiple testing [29], [30]. In contrast, although both the CT and AC groups improved in the Language measure (COWAT) over the 6 month period (TIME: F(1,91) = 7.92; *p* = .006), the AC group achieved a greater effect size (*g* = .50) than the CT group (*g* = .28; TIME x GROUP: F(1,91) = 4.41, *p* = .04). This latter finding may reflect the largely language-based nature of the AC activity in the form of active listening and comprehension practised in each session, whereas the CT intervention loaded on language only 20% of the time, with only one out of five activities containing a language component. This finding also remained significant after Bonferroni correction. Both these effects are illustrated in **Figure 2**.

#### 6-month follow-up

There were no significant TIME x TRAINING GROUP effects observed 6 months post intervention. There were also no significant long term TIME x TRAINING GROUP effects when analysed by cognitive ability or age stratification.

### Effect of Cognitive Training on Wellbeing

#### 6-month follow-up

**Figure 3:**
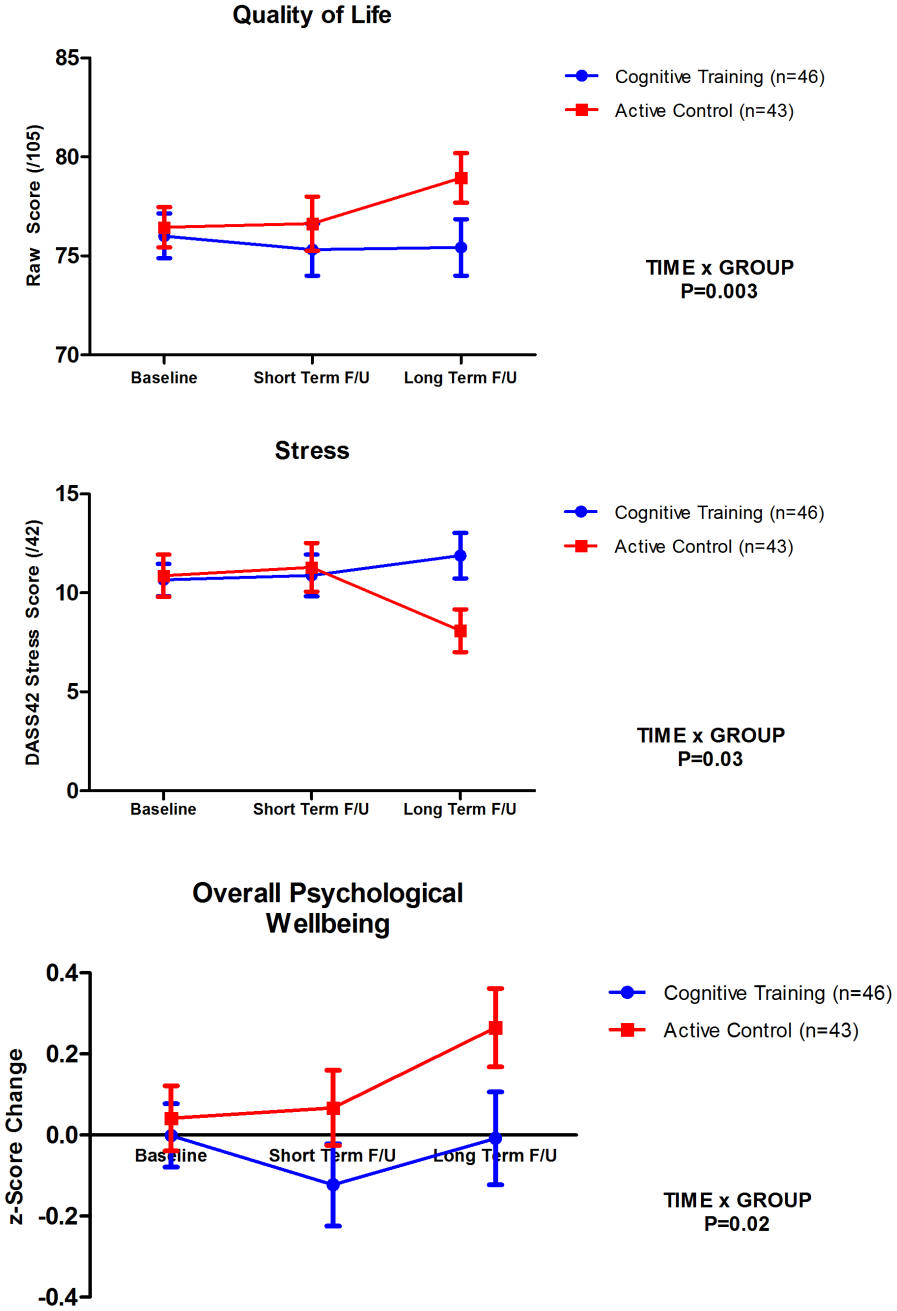
Short and long term effects of CT on Quality of Life, Stress and Overall Psychological Wellbeing. Error bars represent SEMs. P-value is for TIME x TRAINING GROUP interaction at the long term follow up point.

There were no significant TIME x TRAINING GROUP differences on measures of wellbeing immediately after the completion of training. However, significant TIME x TRAINING GROUP differences were found in two wellbeing variables at long term follow up. Unexpectedly, these benefits occurred in the AC rather than the CT intervention. Those in the AC group experienced a significant increase in their self-reported Quality of Life (*g* = .34) compared to the CT group (*g* = −.15; *p* = .003) and stress levels also declined significantly for the AC group (*g* = .22) but increased for the CT group (*g* = −.19; *p* = .03). However, this effect on stress was no longer significant after Bonferroni adjustment. Overall, Psychological Wellbeing improved for the AC group (*g* = .32) but not for the CT group (*g* = −.06; *p* = .02). These findings are illustrated in **Figure 3**. The concordance of these findings over a number of wellbeing measures suggests that our AC condition may have had a positive impact on the self-reported wellbeing of this working sample.

### Effect of Cognitive Training on Productivity

#### On completion of training

There were no significant TIME x TRAINING GROUP differences on various measures of productivity over the short term. A complete participant data set comprised data for each of the nine productivity variables. For employer organisational reasons there were few participants who had all nine pieces of information available at baseline, short term and long term follow up. Using imputation methods to account for the 25% of missing data at short term follow up did not alter our findings.

#### 6-month follow-up

Overall long term productivity effects were inconclusive due to large amounts of missing data (61% missing). After use of imputation techniques, no significant productivity outcomes were observed.

## Discussion

The unqualified use of CT in the work environment amongst healthy adults found no support in this study, even when levels of cognitive capacity and age group were taken into consideration. In terms of wellbeing, significant positive changes were reported over the long term, however, this was observed in the AC rather than the CT intervention. It is possible that this alternative form of mental activity may have a place in the promotion of wellbeing amongst white collar workers, but will require further investigation. Productivity outcomes were difficult to interpret given the large amount of missing data due to inconsistent collection and reporting through the workplace’s organizational systems. A more robust, externally administered and validated tool may be required for future studies intending to evaluate the productivity impact of workplace interventions.

Whilst the impact of CT on cognition may be dose-dependent [31], [32], the ‘dose’ has yet to be determined. So far, the literature suggests that at least four weeks of daily exercises are necessary to enhance cognition in any enduring way in non-aged populations, presuming that the training program is effective at the outset [33], [34]. Lustig et al (2009) conducted an extensive review of studies investigating the effects of various interventions on the cognitive ageing process in healthy older adults [31]. Training dose varied widely, ranging from just one session to 40 hours of training over eight weeks. A meta-analysis of studies of healthy adults suggested that persistent protective benefits required at least a two to three month training program [17]. Our study applied a realistic dose based on the literature (16 hours, 20 minutes three times a week) however it was spread over 16 weeks due to workplace restrictions. This ‘dilution’ of training may be one reason for the limited effects we observed and so the effects of more concentrated and extended doses should be investigated. Other challenges and limitations worthy of note include measurement of cognitive change in a cognitively intact sample, recruitment and retention of participants over a longitudinal study and access to accurate and complete productivity data.

Quite unexpectedly, our AC, which involved viewing an extensive series of short National Geographic documentaries, appeared to have an enduring and positive impact on a number of wellbeing measures. The potential impact of simply taking ‘time-out’ breaks during the work day has recently gained support. In fact, work day breaks have been shown to counter effects of fatigue and actually increase productivity [35]. The nature of any working-day break task also has important implications for the recovery process. Trougakos and Hideg (2009) distinguish between ‘respites’ and ‘chores’ [36]. Respite activities are low effort or preferred by choice, which by their nature allow individuals to restore their personal resources for future work effectiveness. Chores are by contrast non-preferred activities that deplete the individual’s personal resources. Our AC may have been perceived as a respite-type break due to the non-work related content and lack of associated performance pressure, a view supported by qualitative feedback from this group. Further research is required to replicate and understand the nature of this serendipitous finding.

Overall, this trial provides little support for the material benefit of CT to workers in roles of moderate cognitive complexity [37]. On the other hand, new and unexpected evidence was found for the idea that respite-type breaks during work hours can benefit workplace mental wellbeing. For employers, attempting to improve their employee’s cognitive resources may provide limited returns, whereas attention to their mental health and wellbeing could potentially result in improved work performance [7], [8]. Workplace interventions targeting mental health, rather than cognition alone, may help employees and employers achieve a more productive work environment.

## Supporting Information

Checklist S1 **CONSORT Checklist.**
(DOC)

Protocol S1 **Trial Protocol.**
(DOC)

Supporting Information S1 **Supporting Information.**
(DOCX)
