# Supplementary material for: Putting Brain Training to the Test in the Workplace: A Randomized, Blinded, Multisite, Active-Controlled Trial
Source: PLoS One. 2013 Mar 28;8(3):e59982. doi: 10.1371/journal.pone.0059982 (PMC3610917; doi:10.1371/journal.pone.0059982)
Supplement: Protocol S1 — Trial Protocol. (DOC) [file pone.0059982.s002.doc]

Trial Protocol

Version: 25 July 2010

**Full Trial Title**

Boosting the Cognitive Capacity and Mental Wealth of Working Australians

**Short Title**

National Mental Wealth Study

**Chief Investigator**

Dr Michael Valenzuela (School of Psychiatry UNSW)

**Associate Investigators**

Ms Cate Borness (PhD student, School of Psychiatry UNSW)

AProf Judy Proudfoot (School of Psychiatry UNSW)

Dr John Crawford (Statistical consultant, School of Psychiatry UNSW)

**Trial Funding Source**

The Brain Department Pty Ltd: Ms Wendy Hand and Ms Susan Miller (Co-directors)

**Trial Registration**

Australian and New Zealand Clinical Trials Registry ACTRN12610000604000

Aims and Hypotheses

Cognitive brain training involves repeated exercise on problems that target specific cognitive domains. This project aims to test *for the first time* whether *computerized* cognitive brain training can lead to real-world psychological and workplace outcomes in *healthy, working-age individuals*. Through the development by The Brain Department of a new online delivery system for cognitive brain training in the workplace, we wish to specifically test whether computerized cognitive brain training can:

1. Lead to increases in cognitive abilities that are vital to effective and efficient workplace performance
2. Lead to increases in positive-psychological measures of subjective wellbeing and quality of life
3. Lead to increases in objective measures of workplace productivity

Background

**National Mental Wealth**

The Foresight Project on Mental Capital and Wellbeing ^1^ is a landmark UK-based study that used state-of-the-art scientific evidence to examine the socioeconomic challenges and opportunities that lie ahead for developed nations in the next 20 years. Completed in 2008 after two years of research and involving more than 450 experts and stakeholders, this report is now being used to inform policy makers around the world. It identifies two critically important concepts for the ongoing socioeconomic success of developed nations: Mental Capital and Mental Wellbeing. ***Mental Capital*** includes people’s cognitive abilities, emotional intelligence, social skills and resilience in the face of stress ^2^. ***Mental Wellbeing*** is a more dynamic concept, referring to an ability to develop one’s potential, build positive relationships and work productively. Mental capital and wellbeing are closely connected throughout the lifespan, and at a population level, sum to produce a nation’s ***Mental Wealth***. In an overview report of the Foresight Project published in *Nature*, the lead authors underline the broad significance of these concepts to both economic and social development: “(Mental Wealth) not only has a significant effect on its economic competitiveness and prosperity, it is also important for mental health and well-being and social cohesion and inclusion.” ^2^ (p.1057).

**Population Ageing is a Threat to our National Mental Wealth**

The single greatest threat to Australia’s Mental Wealth is the ageing of our population. Demographers predict that by mid-century, one out of every five persons – 6 million persons - will be 60 years or older ^3^. Given that advanced age is the single greatest risk factor for dementia and cognitive decline ^4^, there are profound implications for our National Mental Wealth. For example, the risk for dementia doubles every 5 years after the age of 60, to include one-in-four individuals over the age of 80 ^5^. Beyond the dramatic rise in diagnosed dementia with age, advancing years are also associated with significant decrements in cognitive capacity. Large cross-sectional and longitudinal studies show that for every 10 years beyond 20 years of age, average memory performance declines by about 8% ^6^. Moreover, problem solving ability (i.e., frontal-executive function) declines by 7% per decade, and ability to focus and concentrate (i.e., attention) by 8% per decade ^7^. These trends are summarized in **Figure 1**.

**Figure 1 (next page)**. *Cognitive change across the lifespan shows that mental abilities such as mental speed, memory and attention decline steadily from the early 20s (from Park & Reuter-Lorentz 2009^8^).*

The median age for all countries is currently 29 and will rise to 38 by mid-century ^9^. In Australia, the median age is already 36.9 years (Australian Bureau of Statistics, 2008) and will rise by a proportionally higher rate than the rest of the world. As the median age of the ‘typical’ Australian rises inexorably higher, this will therefore be associated with a general fall in cognitive capacities. Logically, given cognitive capacity is one of the best predictors of work productivity ^10^, gross national productivity may also be under threat (assuming the status quo).

A recent special report in *The Economist*, highlights the magnitude of dangers inherent in the ageing population ^11^:

“When the IMF earlier this month calculated the impact of the recent financial crisis, it found that the costs will indeed be huge: the fiscal balances of the G20 advanced countries are likely to deteriorate by eight percentage points of GDP in 2008-09. But the IMF also noted that in the longer term these costs will be dwarfed by age-related spending. Looking ahead to the period between now and 2050, it predicted that ‘for advanced countries, the fiscal burden of the crisis (will be) about 10% of the ageing-related costs’. The other 90% will be extra spending on pensions, health and long-term care”.

***Overall, our aggregate mental capital and hence National Mental Wealth face a severe challenge due to the ageing of the population.***

**Opportunities to Boost Mental Wealth**

A number of strategies for promoting and boosting National Mental Wealth have been proposed The Foresight Project identified three key strategies directly relevant to cognitive capacity, work productivity and mental wellbeing (quotes taken from Beddington et al 2008 ^2^):

1. Boosting brain power: “There is huge scope for improving mental capital through different types of intervention”
2. Learning throughout life: “This can have a direct effect on mental health and well-being across all age-groups”
3. Changing Workplace Practices: “Workers’ mental well-being is an important factor when attempting to improve the mental capital of economies and societies”.

Computerised Cognitive Brain Training (CCBT) integrates into these strategic recommendations and represents a practical and potentially effective way of boosting National Mental Wealth.

**What is Computerised Cognitive Brain Training (CCBT)?**

Definitions

In our critical review we defined ***cognitive brain training*** as composed of four critical elements: i) *repeated practice, ii) on tasks with an inherent problem*, iii) using *standardised* tasks, and iv) that target *specific cognitive domains* ^12^. Cognitive brain training research has traditionally been implemented via personally-supervised, paper-and-pencil cognitive exercises ^13^. ***Computerised Cognitive Brain Training*** (CCBT) is, however, a relatively new technological solution to delivering cognitive brain training in a more effective, reliable and cost-effective manner ^12^. A major strength of computer-based interventions is that they enable tailoring for users’ individual differences. For example, algorithms can set the initial level of task difficulty with reference to the individual’s baseline competency, and then gradually increase task difficulty in a customized fashion, in effect providing an individualized intervention. These features also allow effective control of ceiling and floor effects, which theoretically may be a key ingredient for successful cognitive exercise regimes. In addition, computer-based interventions enable the unobtrusive real time monitoring of cognitive performance, the standardization of intervention and potentially the reduction of personnel and implementation costs. These features increase the reliability, operationalization and feasibility of CCBT for both scientific trials and applied implementation.

**Effectiveness of CCBT Across the Lifespan**

During Childhood

A recent report at the British Science Festival of the use of CCBT in a group of children with learning impairment provoked worldwide media attention ^16^. This study found that CCBT for 8 weeks resulted in measurable increase in general intelligence as well as improvements in academic attainment. Hence, even during childhood CCBT may be able to augment cognitive capacities and day-to-day function.

During Adulthood

The largest body of research for the effectiveness of cognitive brain training comes from the schizophrenia literature, a disorder which predominately affects young adults. One of the most problematic long-term issues is negative symptoms, including cognitive deficits. A meta-analysis of 26 randomised controlled trials of cognitive brain training in individuals with long term schizophrenia found that overall there was consistent evidence that such training could lead to enhanced cognitive abilities ^17^. Overall, the effect size on cognition in the trained group compared to control groups was 0.41. Interestingly, a study which directly tested a CCBT approach using a commercial brain training product found not only improved cognitive outcomes, but also decreased time to re-enter the workforce. Hence, CCBT can potentially not only affect outcomes directly relevant to the day-to-day function, but also ameliorate some of the socioeconomic consequences of the disease ^18^.

During Later Life

We have systematically reviewed the effectiveness of cognitive brain training in those healthy individuals over 65 years of age and found that there is consistent evidence for a measurable improvement in cognitive abilities ^19^. Overall, the magnitude of the relative effect size was strong (+1.09SD), including studies that have used a CCBT strategy. A recent report has also found that use of a CCBT product can not only lead to significant improvements in cognitive capacities, but also help prevent the onset of depressive symptoms ^20^.

***There is therefore strong evidence to suggest that in clinical and ageing populations, CCBT can lead to improved cognitive capacities, improved mental health outcomes and also ‘real world’ socioeconomic benefits. To our best knowledge, however, there has yet to be a scientific investigation of CCBT in cognitively-intact, healthy working individuals. This is therefore the overarching aim of this project***

**How could CCBT improve work productivity?**

Workplace productivity is a complex construct, spanning the general perspective of how effectively an organization uses its resources to achieve goals ^21^, to more concrete calculations of financial outcomes with respect to the cost of achieving those outcomes ^22^. Important predictors of workplace productivity include intelligence and cognitive abilities ^10^, and personality characteristics such as conscientiousness and integrity ^23^.

Given the efficacy of CCBT in clinical and normal ageing populations in increasing cognitive performance, improving mood symptoms and decreasing time to re-employment (as reviewed above), ***we predict that similar improvements are also possible in healthy working Australians – with an overall positive effect on workplace productivity***. In terms of fundamental neurobiological mechanisms, we have proposed a framework in which these changes are reliant on a range of neuroplastic adaptations induced by CCBT, including biochemical, molecular, cellular and cortical network changes throughout different regions of the brain ^24,25^.

**What to Measure? The Generalization Hierarchy**

We have previously criticized studies of cognitive brain training for simply focusing on measurable gains on the same trained task, a predictable effect of trivial significance ^19^. Instead, in the project we propose to measure the effectiveness of CCBT on the full range of outcomes with scientific, business and health relevance. Using the principle of a hierarchy of generalized outcomes (see **Figure 2**)^19^, we will be specifically examining the effect of workplace CCBT on:

**1. Cognitive Capacities**: the full range of cognitive abilities will be tested before and after CCBT, with careful selection of cognitive tests that are NOT the same as the trained tasks, and assessment of general cognitive performance.

**2. Mental Wellbeing**: psychometrically valid tests of workplace satisfaction, stress, depression and anxiety symptoms, as well as positive psychological concepts such quality of life and self-esteem

**3. Workplace Productivity**: Encompassing both time to complete activities, as well as quality based measures.

**Figure 2.** *The Hierarchy of Generalization*

**Significance**

This is the first RCT of CCBT in healthy working age-individuals where training has been delivered directly to workers’ computers. This trial is furthermore designed to test the ‘real world’ applicability of CCBT by examination cognitive transfer of effect, and whether there is any generalization of effect of mental well being and workplace productivity.

Outcomes from this study will therefore be of high interest to not only researchers, but also business leaders, workers, media and policy-makers. The scientific outcomes will not only lead to peer-review publications in leading general psychology and science journals, but also critically inform design of future scientific studies of CCBT in healthy working populations.

**About the Funding Source and Spark! CCBT product**

In the US, CCBT already accounts for a $225million market (www.sharpbrains.com). There are an increasing number of ‘brain training’ products; a list on www.sharpbrains.com identifies more than 20. One of problems and challenges for the industry is that it is currently unregulated and being marketed directly to consumers. There are already several instances of unrealistic and unsubstantiated claims, and the attendant risk of unrealistic expectations by consumers.

A subset of the commercial companies is, however, interested in validating their products by sponsoring scientific research. PositScience is one such company with published results ^14^, as well as Lumosity.com who have a number of clinical trials underway.

HappyNeuron Inc is a US-based company that has also published a number of papers testing the effectiveness of their product ^15^. Our Partner Organisation – The Brain Department Pty Ltd – holds exclusive Australian & New Zealand rights over a suite of HappyNeuron Inc CCBT exercises for commercialization in the workplace. The Brain Department has also independently developed ‘*Spark*!’ proprietary software that fully manages the IT implementation, tracking and reporting of these CCBT exercises in the workplace.

Study Methods

**Sample**

Power calculations (see below) suggest 220 subjects will be required; these will be volunteers aged 18-65 years to reflect the general working population and employed for a minimum of 6 months at the Australian Taxation Office (ATO), a national public sector organisation with offices in all state capital cities. Volunteers will be sought from the Debt Capability division, being one of the largest in the ATO, where staff have similar productivity indicators (average handling time, quality, conversion rates) as well as daily use of their own personal workstation.

Subjects will be screened for exclusion of major psychiatric history or current illness by self report. Current illness includes medical treatment for major depression , schizophrenia or bipolar disorder. History includes three or more episodes of major depression or bipolar requiring medical treatment in the last 5 years, or past suicide attempt. Current or history of drug or alcohol abuse is also exclusionary. Participants will not be engaged in any other form of brain training, and they must be at work during the 16-week intervention training period with no more than three weeks of consecutive leave taken during this time.

The ATO has given permission for volunteers in this study to take 20 minutes of their regular work time (i.e., not lunch or break times) three times a week for the purpose of this research for the period of the study. Volunteers will be recruited from, and complete testing and training at, 7 different ATO sites: Sydney CBD, Parramatta, Melbourne CBD, Dandenong, Adelaide CBD, Brisbane CBD, Brisbane (Upper Mount Gravatt). A rolling recruitment approach will be used to make maximal use of resources. Confidentiality of participants’ individual results and randomization status will be guaranteed, except for productivity measures which are provided by the employer. The employer will only have access to aggregate results at the completion of the research. Human Ethics Committee approval for this study was provided by UNSW (HREC 10103).

**Design**

A randomized, active controlled, single-blind, multi-centre intervention trial with longitudinal follow-up will be conducted. CONSORT criteria will be fully implemented in design and reporting. Subjects will be recruited, informed consent given, screened and then undergo a baseline set of assessments, followed by randomization (by computer generated sequence) to the CCBT arm or the active control arm on a 1:1 ratio. Subjectwise randomization will occur across all sites. Concealment of randomisation was achieved by central off-site administration and communicated via email. CCBT training will then commence for 16 weeks (4 months), followed by immediate proximal follow-up, and then longitudinal follow-up 6 months later (i.e., approximately one year since start of trial). The overall sequence of events is provided in **Figure 3**.

**Interventions**

CCBT: those randomized into this condition will complete 3 sessions a week, of 20 minutes each for a total of 16 weeks. CCBT will be conducted during normal work hours and delivered via the ‘*Spark*!’ online system directly to the subject’s normal work computer. This system is propriety software of The Brain Department.

The specific cognitive exercises in the CCBT program are equivalent to HappyNeuron Inc’s validated suite of 36 training options (under Australian and New Zealand exclusive license to The Brain Department). These exercises target the cognitive domains of memory, attention, language, executive functions and visuospatial abilities. A multi-domain approach is likely to be maximally effective as suggested by our recent systematic review ^19^. During each session, subjects will practice a number of exercises from across a range of cognitive domains, and gradually be challenged by exercises of greater complexity and cognitive demand during the course of the 4-month period in an individualized fashion prescribed by the system’s in-built algorithms.

Active Control: those randomized to this condition will view a series of educational National Geographic videos at their work station and answer related multiple choice questions delivered via an online survey. Frequency and duration of computer-based audiovisual stimulation matches the CCBT intervention and cognitive load has been minimised.

**Randomized**

**Volunteer**

**CCBT**

20 mins per session,

3 sessions a week, for 4 months

**Active Control**

20 mins per session,

3 sessions a week, for 4 months

**Proximal Follow-up**

Cognitive Tests

Mental Well being scales

Workplace Productivity review

**Longitudinal Follow-up**

Cognitive Tests

Mental Well being scales

Workplace Productivity review

**Baseline Assessment**

Cognitive Tests

Mental Well being scales

Workplace Productivity review

**Active Control**

Option to complete CCBT

**Figure 3.** *Trial Design*

**Outcome Measures**

As mentioned in Background, we will test CCBT rigorously against three ‘real-world’ sets of primary outcome variables. Background variables controlled for include age, gender and site. Outcome measures will be collected and supervised by a lead research organizational psychologist (C.B) who will remain blind to the training status of subjects.

1. Cognitive Capacities

Subjects will undergo a series of neuropsychological tests that will take no more than 1 hour, given the ATO’s preference to keep testing sessions brief. These will be administered in person by trained research psychologists and include:

1. Computer-based adaptation of WAIS-3 Matrix Reasoning ^36^: Form A and B will be counterbalanced at baseline and follow-up
2. A subset of tests taken from *Mindstreams*^35^*,* a validated computer-based neuropsychological assessment system: Verbal Memory, Non Verbal Memory, Staged Information Processing Speed, Visual-Spatial Orientation, Stroop Interference,
3. A subset of tests taken from *CogScreen*^34^, a validated computer-based neuropsychological assessment system: Visual Sequence Comparison, Divided Attention Parts I and II
4. Controlled Oral Word Association (COWAT): Form A (FAS) will be administered at baseline and Form B (CFL) at follow-up ^33^

All tests each have alternate forms to minimize retest effects.

*Cognitive Primary Outcome Measures:* The following theory-based combinations of test outcomes will be calculated in the form of a sum of standardized z-scores to produce summary domain scores:

1. Memory: Verbal memory and Non-verbal memory
2. Attention: Divided attention Part I and Part II
3. Visual Spatial: Visual Spatial Orientation
4. Language: COWAT
5. Disinhibition: Stroop
6. Reasoning and problem solving: Matrix
7. Speed: Staged Information Processing Speed, Visual Sequence Comparison.

In addition, two *General Cognitive summary scores* will be calculated:

1. Average of domain scores
2. First principal component of baseline cognitive tests results

*Cognitive Secondary Outcome Measures:* Each individual cognitive test score.

2. Workplace Mental Wellbeing

We have operationalised workplace mental wellbeing to include 2 dimensions. Each is measured by a combination of internationally accepted and validated scale with known test-retest reliability and previous use in intervention trials.

1. Job Satisfaction:
   1. Overall job satisfaction scale ^28^ for positive appraisals
   2. Intention to quit scale ^29^ for negative appraisals
2. Psychological wellbeing:
   1. Relative absence of Psychological Distress: this will be measured by the Depression, Anxiety and Stress Scales (DASS^27^).
   2. Self-Esteem: Professional self esteem scale (as modified by Proudfoot et al 2009 ^30^)
   3. Quality of Life: the Quality of Life Scale (QOLS ^31^) and Ryff’s Scales of Psychological Wellbeing (SPWB^32^)

These self report scales will be administered via email and online survey at baseline, proximal follow-up and longitudinal follow-up.

*Primary Outcome Measures:*  job satisfaction and psychological wellbeing will be calculated as the sum of standardized z-scores.

*Wellbeing Secondary Outcome Measures:* Each individual wellbeing score.

3. Workplace Productivity

In collaboration with the ATO we have identified the following productivity indicators:

1. average handling time
2. conversion rates
3. work quality ratings

These will be provided by the ATO for each individual at baseline (on the basis of the 6 months performance before starting the study), at proximal follow-up and at longitudinal follow-up (on the basis of approximately 12 months since commencement of the study).

*Productivity Primary Outcome Measure*: Overall productivity will be calculated as the sum of standardized z-scores across each productivity measure.

*Productivity Secondary Outcome Measures*: Each individual productivity measure.

**Statistical Analysis**

Analysis will be by intention-to-treat using a repeated measures mixed modeling approach. Primary outcomes will be considered separately within each set (i.e., cognitive, wellbeing and productivity). Both univariate and multivariate analyses accounting for multiple comparisons and background variables will be conducted. Absence of any differences on primary outcome measures between ATO work location would lead to collapsing data across sites, otherwise SITE will be entered in the model as a covariate. For each analysis, within-subject change will be tested (TIME), followed by between GROUP differences, and then the main analysis will test the interaction between TIME X GROUP. Statistical significance will be set at alpha = 0.05. Treatment of missing values will depend on the extent and randomness of incomplete data.

Planned Subgroup analysis

A planned subgroup analysis of individuals categorized as high versus low on baseline cognitive testing (by median split of cognitive performance) will be conducted to determine if outcomes interact with initial level of cognitive competency.

Exploratory analysis

Depending on the nature of the results, exploratory structural equations modeling analyses may be run to determine the nature of any positive effects with respect to interactions between cognitive, wellbeing and productivity outcomes.

Power & Sample Size

Based on pilot study findings, effect size estimates for the domains of Memory and Executive function are presented in the table below. Note that positive effects are the desired direction for accuracy outcomes, and negative effects for response time outcomes. *Relative Effect Siz*e refers the Cohen’s d effect size in CCBT group (Pre-Post mean difference divided by pooled variance) minus the effect size in the control group.

|  |  | **Memory** | Executive Function |
| --- | --- | --- | --- |
| **Cohen's Relative Effect Size *d*** | **Accuracy** | 0.65 | 0.39 |
|  | **Response Time** | -0.51 | -0.99 |
| **Required Sample Size (n per group)** | **Accuracy** | 36 | 102 |
|  | **Response Time** | 66 | 22 |

Given the range of effect sizes observed, the most conservative effect size of 0.39 (on Executive accuracy scores) has been chosen. Controlling power at 0.8, 102 subjects would therefore be required per arm (rounded down to 100). After inflation for an expected 10% attrition rate, this results in an initial sample of N=220, (each arm to comprise n= 110).

References

1. Jenkins, R. & et al. Foresight Mental Capital and Wellbeing Project. Mental Health: Future Challenges. 2008. Government Office for Science.
 2. Beddington, J. *et al.* The Mental Wealth of Nations. *Nature* 455, 1057-1060 (2008).

3. Access Economics. Dementia: Burden of Disease in Australia. 2005.

4. Brayne, C. The elephant in the room - healthy brains in later life, epidemiology and public health. *Nature Reviews Neuroscience* 8, 233-239 (2007).

5. Jorm, A., & et al. The prevalence of dementia: a quantitative integration of the literature. *Acta Psychiatrica Scandinavica* 76, 465-479 (1987).

6. Wechsler, D. *Wechsler Memory Scale - Third Edition. Administration and Scoring Manual.* The Psychological Corporation. Harcourt Brace, San Antonio., (1997).

7. Wechsler, D. *Wechsler Adult Intelligence Scale - Third Edition. Administration and Scoring Manual.* The Psychological Corporation. Harcourt Brace, San Antonio., (1997).

8. Park, D. & Reuter-Lorenz, P. The Adaptive Brain: Aging and Neurocognitive Scaffolding. *Annual Review of Psychology* 60, 173-196 (2009).

9. United Nations Population Division. World Population Prospects: The 2006 Revision. 2007.

10. Ree, M. & Earles, J. Intelligence is the Best Predictor of Job Performance. *Current Directions in Psychological Science* 1, 86-89 (1992).

11. A Slow Burning Fuse: A Special Report on Ageing Populations. The Economist . 6-25-2009.

12. Gates, N. & **Valenzuela**, M. Cognitive Exercises and its Role in Cognitive Function in the Elderly. *Current Psychiatry Reports* . (2009) In Press (Accepted 08/04/09).

13. Rebok, G., & et al. Training and maintaining memory abilities in healthy older adults: traditional and novel approaches. *Journal of Gerontology: SERIES B* 62B, 53-61 (2007).

14. Mahncke, H. *et al.* Memory enhancement in healthy older adults using a brain plasticity-based training program: a randomised, controlled study.*Proceeding of the National Academy of Sciences, USA.* 103, 12523-12528 (2006).

15. Croisile, B. Memory Stimulation. Which Scientific Benefits? Which Exercises? *Revue de Geriatrie* 31, 421-433 (2006).

16. Alloway, T. & Alloway, R. The Efficacy of Working Memory Training in Improving Crystallized Intelligence. *Nature Precedings.* hdl:10101/npre.2009.3697.1 (2009).

17. McGurk, S., & et al. A Meta-Analysis of Cognitive Remediation in Schizophrenia. *American Journal of Psychiatry* 164, 1791-1802 (2007).

18. McGurk, S., & et al. A. Cognitive training and supported employment for persons with severe mental illness. *American Journal of Psychiatry* 437-441 (2007).

19. **Valenzuela**, M. & Sachdev, P. Can cognitive exercise prevent the onset of dementia? A systematic review of randomized clinical trials with longitudinal follow up. *American Journal of Geriatric Psychiatry* 17, 179-187 (2009).

20. Wolinsky, F. *et al.* The ACTIVE Cognitive Training Interventions and the Onset of and Recovery from Suspected Clinical Depression. *Journal of Gerontology: Psychological Sciences* 64B, 577-585 (2009).

21. Pritchard, R. *Handbook of Industrial and Organizational Psychology*. Dunnette, M. & Hough, L. (eds.), pp. 443-471 (Consulting Psychologists Press. Palo Alto, CA.,1992).

22. Mahoney, T. *Productivity in Organizations*. Campbell, J. & Campbell, R. (eds.) (Jossey-Bass. San Francisco., (1988).

23. Schmidt, F. & Hunter, J. The Validity and Utility of Selection Methods in Personnel Psychology: Practical and Theoretical Implications of 85 Years of Research Findings. *Psychological Bulletin* 124, 262-274 (1998).

24. **Valenzuela**, M., Breakspear, M. & Sachdev, P. Complex Mental Activity and the Ageing Brain: Molecular, Cellular and Cortical Network Mechanisms. *Brain Research Reviews* 56, 198-213 (2007).

25. **Valenzuela**, M. J. *et al.* Memory training alters hippocampal neurochemistry in healthy elderly. *Neuroreport* 14, 1333-1337 (2003).

27. Lovibond, P. & Lovibond, S. The structure of negative emotional states: comparison of the depression anxiety stress scales (DASS) with the Beck depression and anxiety inventories. *Behaviour Research and Therapy* 33, 335-343 (1995).

28. Warr, P., Cook, J. & Wall, T. Scales for the measurement of some work attitudes and aspects of psychological well-being. *Journal of Occupational Psychology* 52, 129-148 (1979).

29. Guest, D., Peccei, R. & Thomas, A. The impact of employee involvement on organisational commitment and 'them and us' attitudies. *Industrial Relations Journal* 24, 191-200 (1993).

30. Proudfoot, J., *et al.* Cognitive-behavioural training to change attributional style improves employee well-being, job satisfaction, productivity, and turnover. *Personality and Individual Differences* 46, 147-153 (2009).

31. Burckhardt, C. & Anderson, K. The Quality of Life Scale (QOLS): reliability, validity, and utilization. *Health and Quality Life Outcomes* 1, 60 (2003).

32. Springer, K. & Hauser, R. An assessment of the construct validity of Ryff's Scales of Psychological Well-being: method, mode and measurement effects. *Social Science Research* 35, 1080-1102 (2006).

33. Ross, T. P., Calhoun, E., Cox, T., Wenner, C., Kono, W., Pleasant, M. (2007). "The reliability and validity of qualitative scores for the Controlled Oral Word Association Test." Archives of Clinical Neuropsychology 22: 475-488

34. Kay, G. G. (1995). CogScreen Professional Manual. Odessa, Florida, Psychological Assessment Resources.

35. Doniger, G. M. (2010). Mindstreams Product Guide V.2.1.4, NeuroTrax Corporation: 1-46.

36. Wechsler, D. (1997). WAIS-III administration and scoring manual. San Antonio, TX: The Psychological Corporation.
