# Supplementary material for: Putting Brain Training to the Test in the Workplace: A Randomized, Blinded, Multisite, Active-Controlled Trial
Source: PLoS One. 2013 Mar 28;8(3):e59982. doi: 10.1371/journal.pone.0059982 (PMC3610917; doi:10.1371/journal.pone.0059982)
Supplement: Supporting Information S1 — Supporting Information. (DOCX) [file pone.0059982.s003.docx]

# Supporting Information S1

# METHODS

Outcome Measures.

*Cognitive Performance* was measured by the WAIS-IV Matrix Reasoning [[1](#_ENREF_1)], Mindstreams subtests of Verbal Memory, Non Verbal Memory, Staged Information Processing Speed, Visual-Spatial Orientation and Stroop Interference [[2](#_ENREF_2)], CogScreen subtests of Divided Attention [[3](#_ENREF_3)], and the Controlled Oral Word Association Test [[4](#_ENREF_4)]. Parallel versions were used on each testing occasion. Secondary cognitive measures were calculated for Memory, Divided Attention, Disinhibition, Processing Speed, Language, Executive Function and overall Global Cognition using z scores.

*Psychological Wellbeing* was measured by the Depression, Anxiety & Stress Scales [[5](#_ENREF_5)] Job Satisfaction Scale [[6](#_ENREF_6)], Intention to Quit [[7](#_ENREF_7)], Professional Self Esteem Scale (as modified by [[8](#_ENREF_8)]), Quality of Life Scale [[9](#_ENREF_9)] and the Scales of Psychological Wellbeing [[10](#_ENREF_10)]. Secondary wellbeing measures were calculated for Job Satisfaction and Psychological Wellbeing using z scores.

*Workplace Productivity* was measured by performance data routinely collected by the organization, for example, the average time taken to complete an activity, how quickly cases were processed, and work quality ratings. An overall “Level of Contribution” (LOC) rating was calculated by adding all the organizational ratings and an overall “Productivity” rating was calculated using z scores across all performance measures. This data was provided to the researcher at the end of each month during the period of the study.

Procedure. Our sample was recruited through presentations conducted at the employees’ work sites. Written informed consent was obtained from each volunteer before proceeding to baseline testing. By giving Informed Consent, participants were agreeing that they did not meet any of the exclusion criteria. Participants were then sent a personalised online link to their psychological wellbeing surveys to complete prior to attending the cognitive testing session. This same procedure was followed for the Short Term and Long Term follow up testing sessions. Once baseline testing was completed, participants were randomized by an independent researcher using computer generated sequence to the CT or AC arm on a 1:1 ratio. Subjectwise randomization was performed across all sites and concealed by central off-site administration. Participants were then sent their link to either the CT or AC program via email. Monthly motivational emails that included current compliance rates were sent to the full participant group by a third researcher who monitored their completion rates on a regular basis.

Statistical Analysis. Raw data were explored for normality at each timepoint and transformations made depending on the skewness of the data. Background variables controlled for included age, gender, education level and work site. Initial bivariate correlational analyses were conducted to ascertain any relationships between the outcome variables. For each analysis, within-subject change was tested (TIME), followed by between group differences (TRAINING GROUP), and then the main analysis tested the interaction between TIME x TRAINING GROUP. Statistical significance was set at α = .05. Percentage compliance, calculated from the number of CT or AC sessions completed out of a total of 48 sessions, was entered as a covariate. For the cognitive capacity median split analyses, baseline data was entered as a covariate to account for any regression to the mean effects.

Given the amount of missing data in the PPC analysis, secondary analyses were run with a full data set generated through imputation techniques (i.e. ITT analysis). This was done using mean replacement, last observation carried forward and expectation maximisation techniques. Normality of data was again reviewed for all imputed data sets andsimilar skews were found for the imputed data sets as were observed with the PPC data set.

# RESULTS

## Schedule of Testing and Training Sessions

| **SITE** | **T1 TESTING** | **FIRST WEEK OF TRAINING** | **FINAL WEEK OF TRAINING** | **T2 TESTING** | **T3 TESTING** |
| --- | --- | --- | --- | --- | --- |
| Sydney CBD | 26-27 Jul 2010 | 2 Aug 2010 | 15 Nov 2010 | 25 Nov 2010 | 17 May 2011 |
| Brisbane CBD | 2 Aug 2010 | 12 Aug 2010 | 25 Nov 2010 | 30 Nov 2010 | 23 May 2011 |
| Brisbane UMG | 3-5 Aug 2010 | 12 Aug 2010 | 25 Nov 2010 | 1-3 Dec 2010 | 24-25 May 2011 |
| Melbourne CBD | 9-10 Aug 2010 | 19 Aug 2010 | 2 Dec 2010 | 14-15 Dec 2010 | 1-2 Jun 2011 |
| Dandenong | 29-30 Jul 2010 | 2 Aug 2010 | 15 Nov 2010 | 13 Dec 2010 | 31 May 2011 |
| Adelaide CBD | 12-13 Aug 2010 | 19 Aug 2010 | 2 Dec 2010 | 8 Dec 2010 | 8 Jun 2011 |

# REFERENCES

1. Wechsler D, editor (2008) Wechsler Adult Intelligence Scale - Fourth Edition: Technical and Interpretive Manual. San Antonio, TX.: Pearson Assessment.

2. Doniger GM (2010) Mindstreams Product Guide V.2.1.4. Columbus, Ohio: NeuroTrax Corporation. pp. 1-46.

3. Kay G (2007) Cognitive Research Corporation. Florida.

4. Spreen O, Strauss E, editors (1991) A Compendium of Neuropsychological Tests: Administration, Norms and Commentary: Oxford University Press: NY, Oxford.

5. Lovibond P, Lovibond S (1995) The structure of negative emotional states: Comparison of the Depression Anxiety Stress Scales (DASS) with the Beck Depression and Anxiety Inventories. Behavioral Research Therapy 33: 335-343.

6. Warr P, Cook J, Wall T (1979) Scales for the measurement of some work related attitudes and aspects of psychological well-being. Journal of Occupational Psychology 52: 129-148.

7. Guest D, Peccei R, Thomas A (1993) The impact of employee involvement on organisational commitment and 'them and us' attitudies. Industrial Relations Journal 24: 191-200.

8. Proudfoot JG, Corr PJ, Guest DE, Dunn G (2009) Cognitive-behavioural training to change attributional style improves employee well-being, job satisfaction, productivity, and turnover. Personality and Individual Differences 46: 147-153.

9. Burckhardt C, Anderson K (2003) The Quality of Life Scale (QOLS): Reliability, Validity, and Utilization. Health and Quality of Life Outcomes 1:60.

10. Ryff CD, Keyes CLM (1995) The Structure of Psychological Well-Being Revisited. Journal of Personality and Social Psychology 69: 719-727.
